# Ancillary Studies in Determining Human Papillomavirus Status of Squamous Cell Carcinoma of the Oropharynx: A Review

**DOI:** 10.4061/2011/138469

**Published:** 2011-07-03

**Authors:** Richard L. Cantley, Eleonora Gabrielli, Francesco Montebelli, David Cimbaluk, Paolo Gattuso, Guy Petruzzelli

**Affiliations:** ^1^Department of Pathology, Rush University Medical Center, 1850 W. Harrison Street, 570 Jelke Building, Chicago, IL 60612, USA; ^2^University of Rome La Sapienza School of Medicine, Viale Regina Elena 324, 00161 Rome, Italy; ^3^Department of Otolaryngology—Head and Neck Surgery, Rush University Medical Center, 1653 W. Congress Parkway, Chicago, IL 60615, USA

## Abstract

Squamous cell carcinoma (SCC) of the oral cavity and pharynx represents the sixth most common form of malignancy worldwide. A significant proportion of these cases are related to human papillomavirus (HPV) infection. In general, HPV-associated SCC is more commonly nonkeratinizing and poorly differentiated, whereas non-HPV-associated SCC is typically keratinizing and moderately differentiated. Nevertheless, significant overlap in morphology is seen between these two forms of SCC. The purpose of this paper is to highlight the utility of ancillary studies in the establishment of HPV status of oropharyngeal SCC, including p16 immunohistochemistry, high-risk HPV in situ hybridization, polymerase chain reaction, and newer HPV detection modalities.

## 1. Introduction

Malignancy of the oral cavity and pharynx constitutes the sixth most common form of malignancy worldwide [1]. In the US, approximately 36,540 cases and 7880 deaths occur per annum [2]. Greater than 90% of these malignancies are squamous cell carcinomas (SCC) [1]. Alcohol and both smoked and smokeless tobacco use are associated with increased risk of developing malignancy of the oral cavity and pharynx [3]. Studies have found a synergism between heavy smoking and heavy alcohol use, with a reported 30-fold increase in risk. As rates of tobacco use have declined, so have rates of oral cavity carcinoma [3].

More recently, human papilloma virus (HPV) infection has been implicated as a major etiologic agent for SCC development [3–12]. HPV consists of a family of encapsulated DNA virus containing over 100 genotypes [4]. High-risk genotypes, most commonly types 16 and 18, are associated with increased risk of squamous cell carcinoma in a number of locations, including cervix, vulva, anus, and oropharynx [4–8]. In contrast to the declining rates of tobacco and alcohol-associated oral cavity carcinomas, the incidence SCC of the oropharynx is increasing, in particular in the base of tongue and tonsils [3, 6–9]. This increased incidence is thought to reflect an increase in HPV-associated SCC. Patients with HPV-associated SCC tend to be younger, more frequently white, and more frequently male compared to those with non-HPV associated SCC [3]. As with cervical SCC, oropharyngeal SCC appears to be associated with sexually transmitted HPV, as high-risk sexual behaviors, including a high lifetime number of sexual partners and younger age at first intercourse, increase the risk [3, 10].

**Figure 1:**
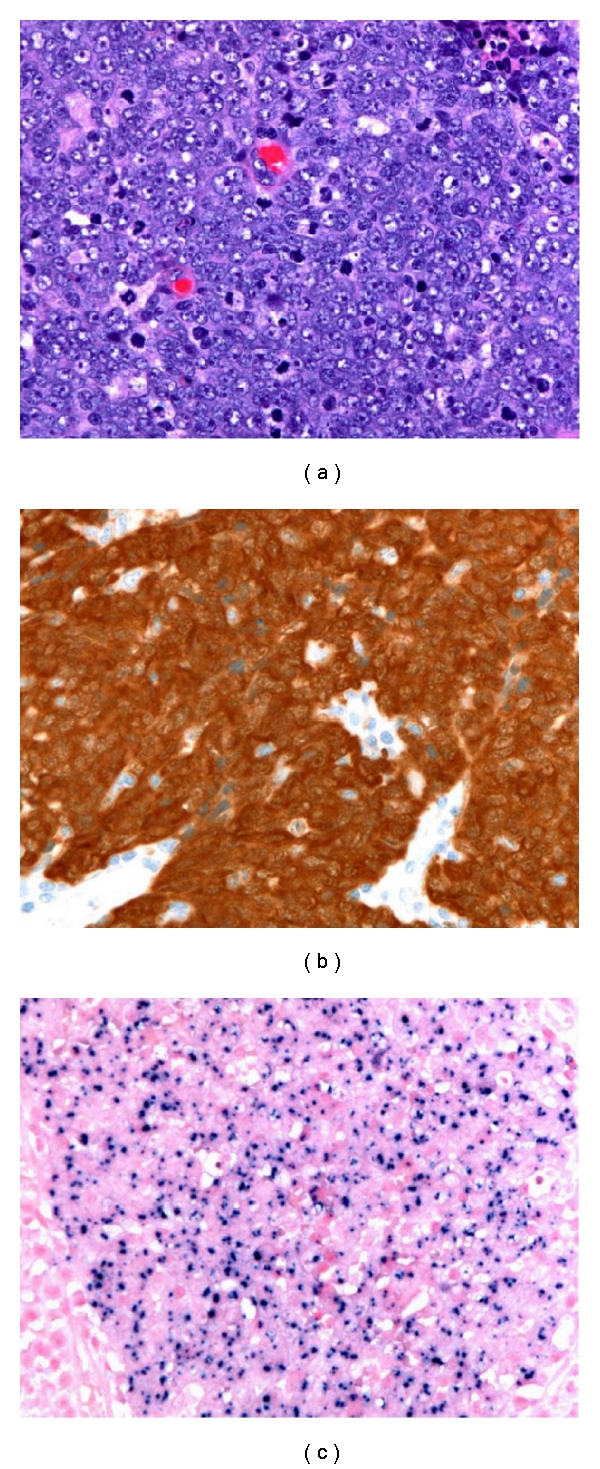
Poorly-differentiated squamous cell carcinoma lacking clear evidence of squamous differentiation (hematoxylin and eosin) (a). In the oropharynx, these are typically HPV-associated neoplasms. Immunohistochemical stain demonstrates diffuse nuclear and cytoplasmic staining for p16 (b), while in situ hybridization highlights the presence of HPV DNA.

**Figure 2:**
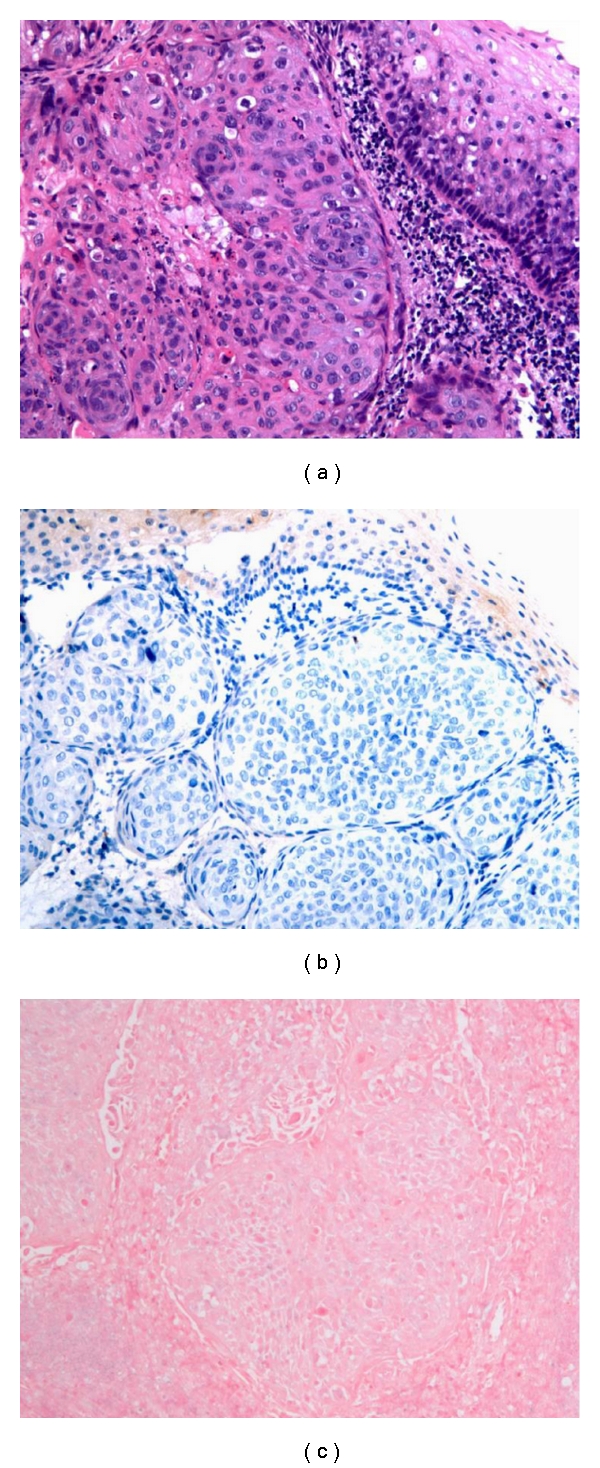
Moderately differentiated squamous cell carcinoma (hematoxylin and eosin) (a) lacking evidence of HPV infection by p16 immunohistochemical stain (b) or in situ hybridization (c). Though well and moderately-differentiated lesions tend to be negative for HPV, and poorly-differentiated lesions are typically HPV positive, there is significant morphologic overlap between HPV positive and negative tumors.

Evidence suggests that there is a causal association between HPV infection and SCC of the oropharynx, with molecular characteristics that distinguish it from non-HPV-related SCC, including alterations of p16 and c-myc expression [13–15]. The protein p16, a cyclin-dependent kinase inhibitor, is frequently utilized as a surrogate marker of HPV infection. Increased nuclear expression of p16 is seen with downregulation of its regulator, Rb protein, as occurs in functional inactivation of Rb by HPV E7 protein [3–5, 16]. Reflecting the differences in pathogenesis, histologic distinctions between HPV and non-HPV-associated SCC are often appreciable. Despite having a better prognosis, HPV associated lesions tend to be nonkeratinizing, poorly differentiated lesions (Figure 1(a)), whereas non-HPV-associated lesions are generally moderately differentiated and keratinizing (Figure 2(a)) [3, 4, 13, 17]. Nonetheless, significant overlap is seen, and both HPV and non-HPV associated tumors frequently demonstrate intermediate features, such as nonkeratinizing tumors with areas of obvious squamous differentiation [13]. 

Distinction between HPV- and non-HPV-related SCC is important in relation to clinical outcome. A study by Ang et al. found three-year survival rate of 82.4% for HPV positive tumors versus 57.1% for HPV negative tumors [18]. A number of additional studies have demonstrated similar outcomes [8, 11, 13, 19–22]. The effect appears unrelated to the particular treatment regimen, as the prognosis has been better for patients treated with radiotherapy [11, 19], concomitant chemotherapy and radiotherapy [11, 18], and surgery alone [20, 21]. Further, the favorable outcome of HPV-associated SCC calls into question the necessity of aggressive postoperative treatment in these cases [22]. In the future, it is possible that treatment strategies may target specific molecular pathways that differ between HPV and non-HPV-associated SCC, further increasing the importance of this distinction.

Despite the importance of establishing the HPV status of SCC, no consensus has been reached on the optimal way to identify HPV-associated SCC [11]. The focus of this paper is the use of ancillary studies in the distinction between HPV positive and negative SCC, including immunohistochemical (IHC) staining for p16, HPV polymerase chain reaction (PCR) testing, and HPV in situ hybridization (ISH) analysis, and newer techniques that are currently under investigation.

## 2. Immunohistochemical Staining for p16

IHC staining for p16 is frequently used as a surrogate marker of HPV infection. It has the advantage of being easy to perform on formalin-fixed, paraffin-embedded (FFPE) tissue, and monoclonal antibodies against p16 are commercially available. HPV protein E7 binds to Rb, a negative regulator of p16 expression. Thus, HPV infection leads to increased nuclear p16 expression. As a result, IHC staining for p16 has a sensitivity approaching 100% for detecting HPV-associated SCC (Figures 1(b) and 2(b)) [13, 15]. 

However, p16 is overexpressed in a subset of tumors apparently lacking evidence for the presence of HPV DNA [4, 13, 22, 23]. Of note, Chernock et al. found that among cases of nonkeratinizing, poorly differentiated SCC of the oropharynx, p16 positivity by IHC staining was present in 100% of cases compared to just 69% positivity for HPV by in situ hybridization [13]. Among these p16 positive tumors, no difference in overall or disease-specific survival was found between those that were HPV positive and HPV negative [13]. Similarly, Lewis et al. found in a series of 239 cases of oropharyngeal SCC that 187 were positive for p16 by immunohistochemical stain [24]. Among these 187 cases, 26 (13.9%) were negative for HPV by both ISH and PCR (using SPF10-INNO primers). In addition, there was no difference in outcome between the p16 positive, HPV positive tumors and the p16 positive, HPV undetectable tumors [24]. In contrast; however, a recent study by Thavaraj et al. using a different set of PCR primers (GP5+/GP6+) from those in the Lewis study found that only 2 out of 142 (1.4%) p16 positive tonsillar SCC were negative for HPV by both PCR and ISH [25].

It is possible, then, that there is a subset of non-HPV-associated tumors with histologic phenotype, molecular characteristics, and prognosis similar to HPV-associated SCC. The percentage of these p16 positive, HPV negative tumors varies significantly between the Lewis and Thavaraj studies. Whether this represents differences in sensitivities of the HPV tests used or true differences in HPV prevalence in different populations is not definitely clear.

In any event, p16 positivity is a sensitive marker for nonkeratinizing, poorly differentiated yet prognostically favorable SCCs. While p16 may not be a specific marker of HPV infection, it can provide important prognostic information, and future therapies aimed at targeting this pathway of HPV tumorigenesis may well be effective in treating p16 positive, HPV negative SCC.

## 3. High Risk HPV In Situ Hybridization

ISH testing for HPV has the benefit of being the only molecular method allowing for direct identification of HPV in topographical relation to the pathologic lesion in tissue (Figures 1(c) and 2(c)) [26]. Unlike other direct detection methods for HPV that are performed in solutions or on solid supports, ISH occurs in the nuclei of infected cells by way of chromogen or fluorescent labeled complimentary nuclei acid probes against either DNA or mRNA [4, 26, 27]. Dot-like or punctuate positivity on microscopic examination indicates integration of the viral genome into the host cell genome, whereas diffuse staining indicates the presence of episomal DNA [4, 26–28].

Numerous technically validated HPV ISH assays are commercially available, most containing a cocktail of probes targeting multiple types of HPV. Though probes for individual types can be used if subtyping is clinically relevant, HPV subtype 16 is by far the most commonly found in oropharyngeal SCC [5, 7, 10, 26]. The commercially available tests include INFORM HPV, Zytofast HPV probe, HPV OncoTect Test Kit, and GenPoint HPV Biotinylated DNA Probe [26]. These tests have demonstrated similar specificity in HPV detection of cervical specimens [26], but to our knowledge, comparisons of the commercially available tests in HPV detection of oropharyngeal lesions have not been performed.

The most common technical difficulties experienced with ISH are background and an absence of signal [27]. Background, defined as nonspecific binding of a probe to nontarget molecules, can be managed by decreasing the concentration of the probe or optimizing the posthybridization wash [26]. Absence of signal can be related to insufficient protease digestion, denaturing temperatures below 95°C, and an insufficient number of copies of the target DNA in the cell [27–29]. Approximately 10 to 20 copies of the target DNA per cell are required for detection by standard ISH techniques [28, 29].

The sensitivity of the assay is increased by signal enhancement techniques. One such technique is tyramide signal amplification, which has been shown to have a 10 to 100-fold increase on sensitivity [28]. In this system, peroxidase-conjugated streptavidin is applied to DNA-DNA hybridization mixture, followed by incubation with biotinylated tyramide. Peroxidase-conjugated streptavidin is then applied, and lastly, the chromogenic substrate diaminobenzidine is added [29]. Using an enzymatic amplification procedure such as this one allows a low copy number of a nucleic acid sequence to be identified. Such techniques have increased the sensitivity of ISH to the extent that it can detect as little as one to two copies of DNA per cell [4, 29].

Nevertheless, the sensitivity of ISH appears to be less than that seen in PCR analysis, as a metaanalysis by Termine et al. found HPV in 39.9% of cases by PCR compared to 29.8% by ISH [30]. However, due to the nature of the probe for specific viral nucleic acid sequences, ISH is highly specific for HPV infection, markedly more so than p16 immunohistochemical staining [28]. Based on the differences in sensitivity and specificity between tests, some authors have recommended two-tiered systems of HPV detection, such as the use of p16 immunohistochemistry as a screening tool and ISH as a confirmatory test [28, 31].

## 4. Polymerase Chain Reaction Detection of HPV

PCR is a process in which a signal sequence of DNA or mRNA is amplified several orders of magnitude through several rounds of denaturing at high temperature (~95°C), annealing of complimentary oligonucleotide primers at a lower temperature (~55°C), and DNA replication at an intermediate temperature (~72°C) by a heat-resistant DNA polymerase. In theory, it can be used to detect as few as one copy of a DNA sequence, making it a highly sensitive detection assay [4].

Material for PCR can be obtained from FFPE tissue by scraping tissue from a tissue block, digesting, centrifuging, and using the resultant supernatant for PCR studies [32]. In addition, samples can be obtained for direct PCR analysis via fresh tissue from oral biopsies [1]. In general, PCR for HPV is more sensitive on fresh frozen tissue compared to FFPE tissue [27].

PCR has the advantages of being highly sensitive for HPV detection, widely available, and cost effective. However, standard PCR techniques have a number of drawbacks. PCR has lower specificity than ISH and is technically cumbersome to perform [27]. In contrast to ISH, it does not allow distinction between HPV that is present in the neoplastic cells and HPV that is present in surrounding nonneoplastic epithelium or stroma, nor can it distinguish between episomal and integrated HPV DNA [4, 26, 27]. In addition, while primers targeting the conserved L1 region are commonly employed, this region may be deleted during viral integration, potentially reducing the sensitivity [27, 32, 33]. However, Agoston et al. found that PCR directed at L1 was more sensitive than PCR directed at the obligate virulence factor E7 (90.2% compared to 72.5%), suggesting that loss of L1 is not seen in a significant number of cases and thus likely does not have a major influence on sensitivity of HPV detection [33].

Several PCR amplification techniques are commercially available. These PCR screening assays commonly have primers designed to amplify a region of DNA that is present in multiple HPV types (most commonly within the highly conserved L1 gene) [4, 33]. Since most commercially available PCR kits use consensus sequences from multiple HPV subtypes, specific typing is generally not possible through PCR alone. Among the more commonly commercially available primer sets are PGMY09/11, GP5+/GP6+, and SPF10 LiPA [34]. All three target sequences within the L1 gene though they are of varying length (450 basepairs, 140 basepairs, and 65 basepairs, resp.). Targeting shorter stretches of DNA generally results in higher sensitivity on FFPE tissue, as DNA fragmentation often occurs during extraction from the archived tissue [34]. Thus, the GP5+/GP6+ and SPF10 primers are more ideal for use in FFPE tissue from surgical specimens. As noted previously, the Lewis et al. study found HPV positivity by PCR in 86% of p16 positive SCC [24], while the Thavaraj et al. study found HPV positivity by PCR in 99% of p16 positive SCC [25]. The notable difference between these studies was the use of SPF10 primers in the former and GP5+/GP6+ in the latter. Differences in sensitivities between the different primer sets could explain this discrepancy. However, to our knowledge, no study has been done to directly compare the sensitivities of these two primer sets in detecting HPV in oropharyngeal SCC.

The use of real-time PCR has also been assessed in HPV detection. Real-time PCR allows for quantification of target DNA via colorimetric markers that accumulate during PCR amplification, allowing a mechanism of identification of HPV DNA as well as an estimation of viral load [4, 23]. This quantitative approach may allow for identification of more clinically relevant high viral loads, and, when targeted against mRNA, provides evidence of active gene transcription [27]. However, real-time PCR does not differentiate between integrated and episomal DNA [23].

Recent studies have looked into the ability of PCR to distinguish between episomal and integrated HPV DNA. The HPV gene for E2 protein is a common break site prior to viral integration into the host genome [4]. E2 protein is a regulator of E6 and E7 proteins, and its gene disruption results in upregulation of these tumorigenic factors [35]. When E2 is disrupted, PCR with primers designed to amplify the entire E2 gene will fail [4]. Thus, comparing PCR amplification of the E2 gene with a gene known to rarely be disrupted during integration (such as the E6 gene) can suggest whether the viral DNA is integrated or not, as the amplification ratio of E2 to E6 would be lower in integrated HPV compared to episomal HPV [4, 20, 36]. However, HPV DNA breakpoints are known to be variable, so E2 disruption is not necessarily seen in all integrated cases, limiting the sensitivity of this technique [4, 37]. Further, it is known that intact episomal E2 may be present even when integrated E2 is disrupted in cases of SCC of the cervix, potentially further reducing the sensitivity [38].

Finally, there are a number of commercially available assays for the detection of HPV by PCR by reverse transcriptase PCR. These kits target mRNA of the oncogenic E6 and E7 proteins. Thus, they have the advantage of detecting transcriptionally active HPV [4, 26]. It has the disadvantage of being time consuming and technically difficult. Further, performance of reverse transcriptase PCR is generally better on fresh tissue than FFPE tissue [4].

Overall, PCR is a reliable, sensitive marker of HPV DNA. Nonetheless, ISH still has a number of advantages over PCR, including higher specificity, the ability to reliably distinguish episomal from integrated HPV DNA, and the ability to localize HPV to the area of neoplasia.

## 5. Additional Techniques for HPV Detection

Over the past two decades, a technique has been developed for combining PCR and ISH, referred to as PCR in situ hybridization (PISH) [27, 39, 40]. In this case, PCR is performed using typical PCR reagents performed on FFPE tissue slides [27, 39]. The slide is then washed, dehydrated in alcohol, and dried. The PCR products present on the slide are then hybridized with specific DNA probes in the same manner that standard ISH is performed [39]. PISH can be utilized to perform PCR for HPV on intact tissue preparations of SCC followed by in situ hybridization detection, thus combining the sensitivity of PCR with the tissue localization of ISH [27, 39]. Studies looking at HPV detection rates in cervical invasive and in situ SCC have found significantly higher detection rates with PISH compared to ISH alone [39–42]. However, to date, no studies have looked at the utility of PISH in detecting HPV in oropharyngeal SCC.

Another hybridization technique, coined hybrid capture II (HC-II) has been developed and utilized in the detection of HPV. This is an FDA-approved method for HPV detection in cervical pap smears, and studies have demonstrated its utility in demonstrating the presence of HPV in lesions of the cervix and oropharynx [1, 26, 43, 44]. Suspicious lesions in the oropharynx are sampled by brush [1]. DNA is extracted from the exfoliated cells, denatured, and converted to single-stranded form [1, 26]. RNA probes against individual HPV subtypes—typically as a cocktail of multiple high-risk types—are then hybridized in solution [1]. These DNA-RNA hybrids are put in microwell plates coated with anti-DNA-RNA hybrid antibodies. The immobilized complex is then reacted with antibodies conjugated to alkaline phosphatase, and cleavage of an added chemiluminescent substrate is measured by emitted light [1]. The intensity of the light emitted allows for an estimation of the viral load. Chaudhary et al. found increased sensitivity for HC-II compared to PCR in the detection of HPV in oropharyngeal SCC [1]. This test has the advantage of allowing HPV testing without the need for biopsy. However, because the reaction occurs in solution, it does not allow for localization of HPV to a histological area of interest. In addition, the high-risk probe cocktail typically used has been shown to detect at least 28 non-targeted HPV types, including many low-risk HPV types, creating the potential for false positives [26].

While IHC staining against p16 is frequently used as a surrogate marker of HPV, to date, IHC staining against specific HPV proteins has generally not been performed. Nevertheless, the development of IHC stains against the oncogenic E6 and E7 proteins would have a number of potential advantages over other HPV detection methods. It would have the ability to prove that HPV DNA is being expressed and directly demonstrate that important HPV oncogene proteins are present [4]. Development of reliable antibodies against E6 and E7 protein could be an excellent means for HPV detection in the future.

## 6. Conclusion

A number of tests for the detection of HPV in oropharyngeal SCC are available, each possessing its own strengths and weaknesses. At the present time, IHC staining for p16 and PCR for HPV appear to be the most sensitive markers of HPV, while ISH confers the greatest specificity. For most clinical laboratories, the combination of a sensitive test (e.g., p16 IHC) and a specific test (e.g., ISH) allows for the best potential to accurately establish the presence or absence of HPV in a given case of SCC. 

The study by Thavaraj et al. utilizes an algorithm originally developed by Weinberger et al. [25, 45] in which oropharyngeal SCCs are classified first by p16 status and then by HPV status (either by PCR or ISH). In this way, SCCs are categorizing as p16−/HPV− (Class I), p16−/HPV+ (Class II), p16+/HPV+ (Class III), or p16+/HPV− (Class IV). Of note, when PCR was used to assess HPV status, 9% of cases fell into the Class II category (p16−/HPV+) compared to just 1% when ISH was used [25]. Given the fact that HPV PCR is known to lack specificity relative to ISH (89% specificity in the study by Smeets et al.), HPV positivity in the absence of p16 positivity by IHC may represent false positivity [23, 25]. In contrast, when ISH was used to assess HPV status, 11% of cases fell into the Class IV category (p16+/HPV−) compared to just 2% by PCR. Since ISH is known to lack sensitivity relative to PCR (86% sensitivity in the study by Smeets et al.), it is reasonable to assume that some of these Class IV cases represent false negatives [23, 25].

Given the relative frequency of discordant p16 and HPV results (i.e., Class II or Class IV) when using p16 in conjunction with a single HPV test, Thavaraj et al. suggest a three-tiered algorithm. Tumors are still categorized as p16 positive or negative by IHC. Those that are negative are then assessed for HPV by ISH and ultimately categorized accordingly as Class I (p16−/HPV−) or Class II (p16−/HPV+). Likewise, tumors that are p16 positive are initially assessed for HPV by ISH, and if ISH is positive, the tumor is categorized as Class III (p16+/HPV+). However, if the tumor is negative by ISH, a confirmatory PCR test is performed. If the tumor is still negative by PCR, it is classified as Class IV (p16+/HPV−). On the other hand, if the tumor is positive for HPV by PCR, the tumor is considered Class III (p16+/HPV+) despite the negative ISH result.

In their study, this three-tiered approach resulted in just a small minority of cases falling under one of the discordant categories (1% class II, 1% class IV), with concordance of p16 and HPV results in 97% (35% class I, 62% class III). This study suggests that with the three most commonly utilized tests, HPV status can be confidently determined in the vast majority of cases of oropharyngeal SCC.

In the future, more recently applied molecular technologies, such as PISH and HC-II, may offer even more accurate diagnosis of HPV in the clinical laboratory, and development of IHC against important viral proteins may ultimately provide the optimal test for active HPV genomic transcription and translation in SCC. Nonetheless, using current ancillary tests in combination with clinical clues and morphology, such as the presence of nonkeratinizing, poorly differentiated lesions, HPV status can be accurately assessed in the vast majority of cases of oropharyngeal SCC.
